# Effects of perfusion fixation on whole-brain structural connectivity in marmoset: a diffusion MRI analysis

**DOI:** 10.1007/s12194-026-01036-y

**Published:** 2026-04-17

**Authors:** Daisuke Yoshimaru, Tomokazu Tsurugizawa, Naoya Hayashi, Kanako Muta, Shuhei Shibukawa, Hirotaka James Okano, Hideyuki Okano, Junichi Hata

**Affiliations:** 1 Division of Regenerative Medicine, The Jikei University School of Medicine, Tokyo, Japan; 2 Laboratory for Marmoset Models of Brain Diseases, RIKEN Center For Brain Science, Saitama, Japan; 3 National Institute of Advanced Industrial Science and Technology (AIST), Tsukuba, Japan; 4 Faculty of Engineering, University of Tsukuba, Tsukuba, Ibaraki Japan; 5 Graduate School of Human Health Sciences, Tokyo Metropolitan University, 7-2-10 Higashiogu, Arakawa, Tokyo, 116-8551 Japan; 6 Department of Radiology, Tokyo Medical University, Tokyo, Japan; 7 Keio University Regenerative Medicine Research Center, Tokyo, Japan; 8 Faculty of Health Science, Department of Radiological Technology, Juntendo University, Tokyo, Japan

**Keywords:** Structural connectivity, Tractography, Perfusion fixation, Marmoset, Ex vivo MRI, Diffusion-weighted imaging

## Abstract

Ex vivo brain studies are essential in neuroscience research, yet the effects of perfusion fixation on structural connectivity remain poorly understood. We previously demonstrated that axial diffusivity is most sensitive to regional volume changes following fixation at the local tissue level. However, how these microstructural changes affect whole-brain network organization remains unclear. To investigate changes in whole-brain structural connectivity following perfusion fixation, advancing our understanding from local microstructural alterations to macroscopic network-level changes. Twelve common marmosets underwent 9.4T MRI scanning both in vivo and following perfusion fixation (ex vivo). Diffusion-weighted imaging was performed with optimized parameters for each condition. Whole-brain tractography was generated using constrained spherical deconvolution and anatomically-constrained tractography framework. Structural connectivity matrices were compared between conditions using the SIFT2 algorithm for quantitative assessment. We identified 799 connections showing significant differences between in vivo and ex vivo conditions (*p* < 0.05, Bonferroni corrected), 14.9% of all possible connections. The most affected regions included the temporopolar area (37 changed connections), hippocampal formation (32 connections), and mid-temporal area (29 connections). Brain regions with minimal volume changes showed fewer connectivity alterations, indicating that local tissue shrinkage predicts the degree of fixation-induced connectivity changes. Perfusion fixation substantially alters structural connectivity in a region-specific manner that correlates with local volume changes. Temporal and limbic regions are most vulnerable to fixation-induced connectivity alterations. These findings provide crucial reference data for interpreting ex vivo tractography studies and highlight the importance of considering fixation effects in comparative neuroimaging research.

**Supplementary Information:**

The online version contains supplementary material available at 10.1007/s12194-026-01036-y.

## Introduction

Diffusion-weighted magnetic resonance imaging (DWI) has revolutionized our understanding of brain structural connectivity by enabling non-invasive mapping of white matter pathways [1, 2]. Tractography, which reconstructs neural fiber bundles from DWI data, has become an essential tool for investigating brain networks in both healthy and diseased states [3, 4]. A significant proportion of neuroimaging research relies on ex vivo brain tissue, which offers advantages including higher spatial resolution, extended scan times, and the ability to validate findings with histological methods [5, 6].

The transition from in vivo to ex vivo conditions involves perfusion fixation with paraformaldehyde, a process known to cause substantial microstructural changes including tissue shrinkage, altered water diffusion properties, and modified cellular architecture [7, 8]. While previous studies have documented changes in diffusion tensor metrics following fixation [9, 10], most comparative studies between in vivo and ex vivo conditions have utilized different cohorts of animals, introducing confounding factors such as inter-individual anatomical variability [11, 12].

To overcome this limitation, we previously compared individual marmoset brains before and after perfusion fixation within the same animals and demonstrated that axial diffusivity (AD) showed the strongest correlation with regional volume changes among diffusion tensor indices [13]. This finding revealed that local microstructural alterations following fixation are closely linked to tissue volume reduction. However, how these local changes affect whole-brain structural connectivity networks remains unclear. Given that tractography relies on the same diffusion signals that are altered by fixation, connectivity estimates derived from ex vivo data may be substantially affected.

Recent advances in tractography algorithms, particularly constrained spherical deconvolution (CSD) and anatomically-constrained tractography (ACT), combined with the Spherical-deconvolution Informed Filtering of Tractograms (SIFT2) algorithm, now enable quantitative and biologically meaningful assessment of structural connectivity [14–16]. Common marmosets (Callithrix jacchus) serve as excellent models for human brain connectivity studies due to their complex cortical organization and practical advantages for experimental manipulation [17, 18].

The present study investigated how perfusion fixation affects whole-brain structural connectivity, advancing our understanding from local microstructural changes to macroscopic network-level alterations. Using the same 12 marmosets scanned both in vivo and ex vivo, we aimed to: [1] quantify specific changes in structural connectivity following fixation [2], identify brain regions most vulnerable to fixation-induced connectivity alterations, and [3] examine the relationship between local volume changes and network modifications. These findings provide essential reference data for the growing body of ex vivo tractography research and offer important considerations for interpreting connectivity studies using fixed tissue.

## Materials and methods

### Animals

All experimental procedures were approved by the Animal Experiment Committee of the RIKEN Center for Brain Science (H27-2-307) and conducted in accordance with ARRIVE guidelines. Twelve adult common marmosets (Callithrix jacchus; mean age 6.0 ± 2.1 years; 2 males, 10 females) were used in this study. Each animal underwent in vivo MRI scanning followed by perfusion fixation and ex vivo MRI scanning.

### In vivo MRI acquisition

In vivo imaging was performed on a 9.4T BioSpec 94/30 scanner (Bruker BioSpin GmbH, Ettlingen, Germany) equipped with a transmit/receive volume coil (86-mm inner diameter). Animals were anesthetized with isoflurane (1.5–2.5% in oxygen) and positioned supine on the imaging bed. Physiological parameters including heart rate, oxygen saturation, respiration, and rectal temperature were continuously monitored.

Diffusion-weighted images were acquired using a spin-echo echo-planar imaging sequence: TR = 3,000 ms; TE = 25.6 ms; b-value = 1,000 s/mm²; 32 diffusion gradient directions; field of view = 44.8 × 44.8 mm²; matrix = 128 × 128; slice thickness = 0.7 mm; acquisition time = 28.8 min. An additional b = 0 image with reverse phase encoding was acquired for distortion correction.

### Perfusion fixation and ex vivo MRI acquisition

**Fig. 1:**
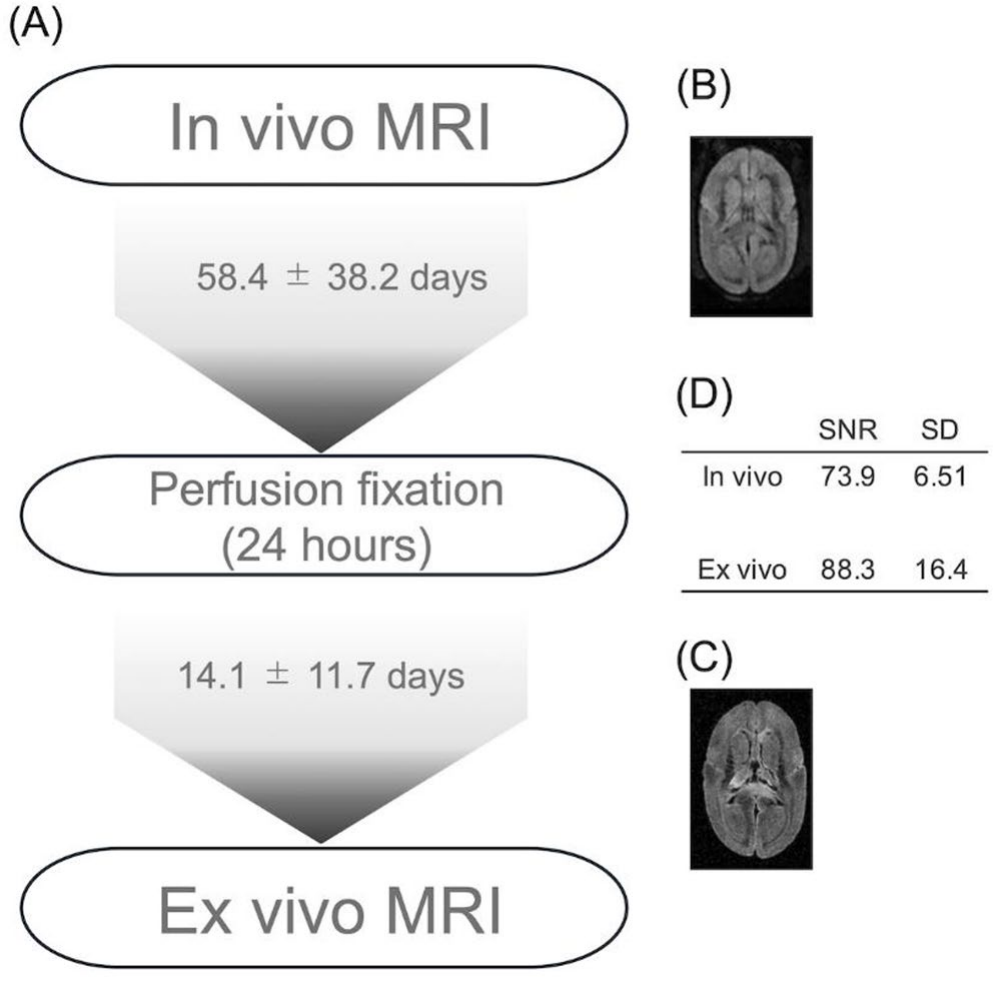
Experimental design, representative brain images, and data quality. **A** Schematic timeline from in vivo MRI to ex vivo MRI showing intervals between procedures. Representative diffusion-weighted images for **B** in vivo and **C** ex vivo conditions (single representative slice each). **D** Signal-to-noise ratio (SNR) measurements for both conditions

Following in vivo scanning (mean interval: 58.4 ± 38.2 days; Fig. 1A), animals were deeply anesthetized with pentobarbital (50 mg/kg, i.p.) and ketamine (10 mg/kg, i.m.). Transcardial perfusion was performed with 4% paraformaldehyde in 0.1 M phosphate buffer (pH 7.4). Brains were extracted from the skull, post-fixed for 24 h at 4 °C, and stored in Fluorinert (FC-72, 3 M) during scanning. Vacuum degassing was performed to minimize air bubble artifacts.

Ex vivo imaging utilized a solenoid coil (28-mm inner diameter) with diffusion-weighted imaging parameters optimized for fixed tissue: TR = 4,000 ms; TE = 28.4 ms; b-value = 3,000 s/mm²; 60 diffusion gradient directions; field of view = 38 × 38 mm²; matrix = 190 × 190; slice thickness = 0.2 mm; acquisition time = 173.3 min. Ex vivo brain diffusion coefficients decrease 2–3-fold compared to in vivo conditions due to temperature changes and tissue microstructural alterations following perfusion fixation [19, 20]. Under ex vivo conditions, the extra-axonal space contains immobile water trapped in microcompartments such as glial cell bodies and vesicles [21]. Therefore, a higher b-value and increased number of gradient directions were employed to optimize signal detection in fixed tissue.

### Image preprocessing

Diffusion-weighted images were preprocessed using MRtrix3 and FSL software packages. Processing steps included MP-PCA denoising to reduce thermal noise, Gibbs ringing artifact removal, and correction for B0 field inhomogeneities, eddy currents, and subject motion using FSL’s topup and eddy tools. Bias field correction was applied using the N4 algorithm [22, 23]. Signal-to-noise ratio (SNR) measurements confirmed sufficient data quality for both conditions, exceeding the QIBA-recommended threshold of 50 [24] (Fig. 1D).

**Fig. 2:**
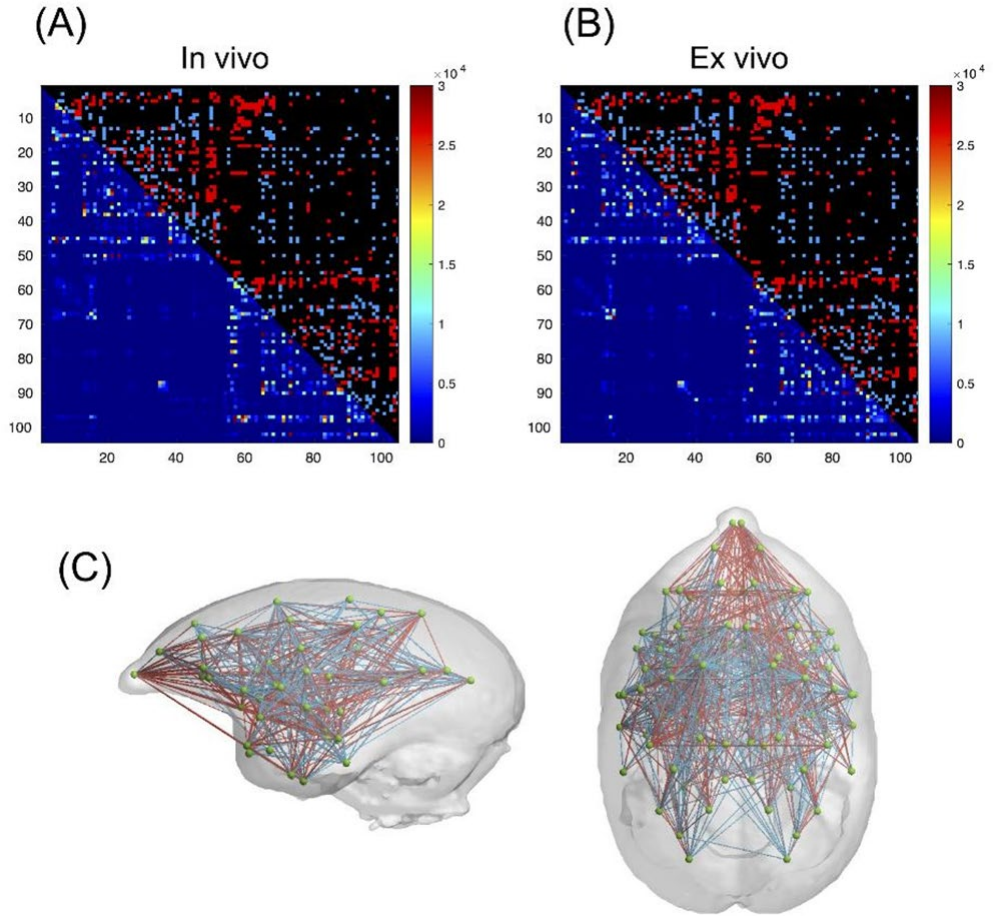
Structural connectivity matrices and connectivity maps. The 104 × 104 connectivity matrices show neural connections for **A** in vivo and **B** ex vivo brains in the lower left of each matrix, where each element represents the mean SIFT2-weighted connectivity across all 12 animals. The upper right shows connections that differed significantly (Bonferroni-corrected paired t-test, α = 0.05; red = increased, blue = decreased, black = not significant). **C** Connectivity maps displaying significant connection differences, with the same color coding as in **A** and **B** (red = increased, blue = decreased)

**Fig. 3:**
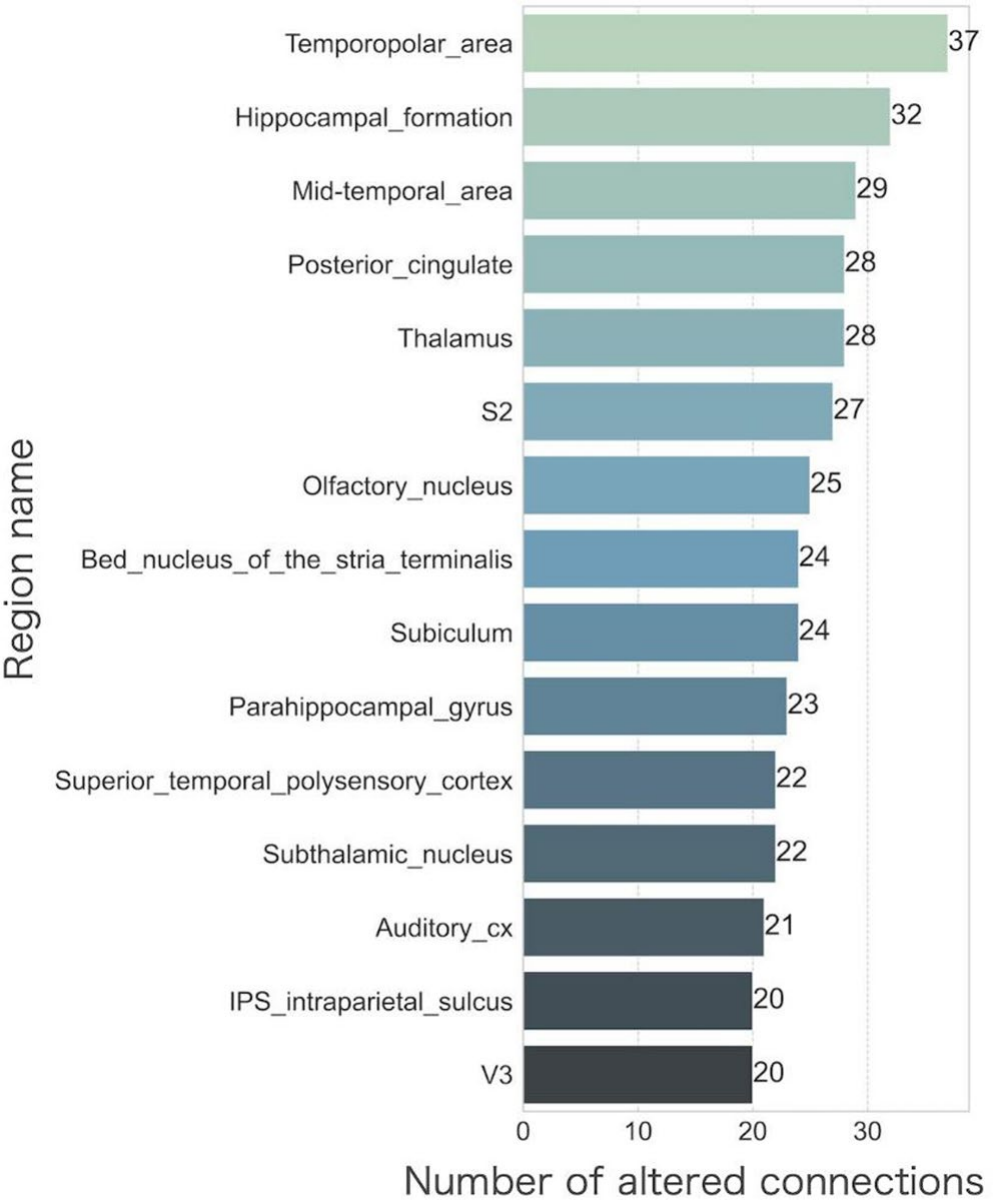
Regional distribution of connectivity changes. The top 15 brain regions with the greatest number of significantly changed connections between in vivo and ex vivo conditions. The x-axis shows the number of altered connections for each region

### Tractography analysis

#### Tissue segmentation

Five-tissue-type (5TT) segmentation was performed to delineate cortical gray matter, white matter, subcortical gray matter, cerebrospinal fluid, and pathological tissue for anatomically-constrained tractography.

#### Fiber orientation distribution estimation

Tissue-specific response functions for white matter, gray matter, and cerebrospinal fluid were estimated using the dhollander algorithm [22]. Multi-shell, multi-tissue constrained spherical deconvolution (MSMT-CSD) was applied to estimate fiber orientation distributions (FODs) throughout the brain [25].

#### Streamline generation

Whole-brain probabilistic tractography was performed using the iFOD2 algorithm with the following parameters: 100 million streamlines; step size = 0.5 mm; maximum curvature angle = 45°; minimum streamline length = 5 mm; maximum streamline length = 250 mm; FOD amplitude cutoff = 0.06. The anatomically-constrained tractography (ACT) framework was employed to improve biological plausibility by incorporating tissue segmentation information during streamline propagation [15].

#### Structural connectivity quantification

Brain regions were parcellated using a marmoset-specific atlas comprising 52 bilateral regions (104 regions total, including both hemispheres; Supplementary Table S1) [26]. Streamline endpoints were assigned to gray matter parcels. The SIFT2 algorithm was then applied to assign biologically meaningful weights to individual streamlines, providing quantitative measures that better reflect underlying fiber density estimated from the diffusion signal [16]. Structural connectivity between each pair of regions was calculated as the sum of SIFT2 weights for all streamlines connecting those regions, yielding symmetric 104 × 104 connectivity matrices for each animal under both in vivo and ex vivo conditions.

#### Statistical analysis

The SIFT2-weighted structural connectivity matrices were compared between in vivo and ex vivo conditions. For each of the 5,356 unique region pairs, paired t-tests were performed across the 12 animals with Bonferroni correction for multiple comparisons (α = 0.05). Connections showing significant differences were identified and mapped onto brain parcellations.

To examine the relationship between tissue shrinkage and network alterations, we quantified the number of significantly changed connections for each brain region. This was correlated with regional volume changes using Pearson correlation analysis. Regional vulnerability was assessed by ranking brain areas according to the total number of altered connections. The relationship between connectivity changes and regional volume changes was also examined using Pearson correlation analysis.

## Results

### Global connectivity changes

Whole-brain tractography analysis revealed substantial differences in structural connectivity between in vivo and ex vivo conditions. Of 5356 possible inter-regional connections, 799 (14.9%) showed significant differences following perfusion fixation (*p* < 0.05, Bonferroni corrected; Fig. 2).

The majority of altered connections showed decreased connectivity strength in ex vivo compared to in vivo conditions (486 connections, 60.8%), while 313 connections (39.2%) showed increased strength. Overall connectivity density decreased from 67.3% in vivo to 58.1% ex vivo.

### Regional distribution of connectivity changes

Connectivity changes were not uniformly distributed across the brain. The top 15 most affected regions are shown in Fig. 3, and the complete results for all 52 brain regions are provided in Supplementary Figure S2. The temporopolar area was most affected, with 37 altered connections, followed by the hippocampal formation (32 connections) and mid-temporal area (29 connections). The posterior cingulate and thalamus each showed 28 altered connections, and the secondary somatosensory cortex showed 27. Other notably affected regions included the olfactory nucleus (25 connections), bed nucleus of stria terminalis (24 connections), subiculum (24 connections), and parahippocampal gyrus (23 connections).

Temporal and limbic structures were consistently among the most affected regions, suggesting particular vulnerability of these areas to fixation-induced network alterations.

### Relationship between volume changes and connectivity alterations

Regions with greater tissue shrinkage exhibited more connectivity alterations. The 10 regions showing non-significant volume changes (piriform cortex, entorhinal cortex, dorsal lateral prefrontal cortex, septal nucleus, gustatory cortex, secondary somatosensory cortex, superior temporal rostral area, posterior parietal area, caudate nucleus, and superior colliculus) had an average of 8.3 ± 4.2 altered connections. In contrast, regions with significant volume reduction showed 18.7 ± 7.4 altered connections on average (*p* < 0.001, unpaired t-test).

### Directional patterns of connectivity change

Decreased and increased connectivity showed distinct spatial patterns (Fig. 2C). The spatial distributions of decreased and increased connections are shown separately in Supplementary Figure S3. Decreased connectivity predominantly affected connections between anatomically distant regions, particularly those involving temporal and frontal areas. This suggests that longer fiber pathways are more vulnerable to fixation-induced geometric distortions.

In contrast, increased connectivity was more commonly observed in connections between neighboring regions and within subcortical networks.

## Discussion

This study demonstrates that perfusion fixation substantially alters whole-brain structural connectivity in a region-specific manner. Nearly 15% of all inter-regional connections showed significant changes following fixation, with connectivity alterations closely associated with regional volume changes. These findings advance our understanding of fixation effects from local microstructural changes to macroscopic network-level alterations, providing critical reference data for interpreting ex vivo tractography studies.

### Mechanisms of connectivity change

The observed connectivity alterations likely result from multiple interacting mechanisms. Perfusion fixation causes non-uniform tissue contraction [7, 8], which alters geometric relationships between brain regions. This mechanical deformation may change the tractography-derived length and curvature of white matter pathways, potentially affecting fiber orientation distribution estimation and subsequent tractography reconstruction. For instance, elongated or more tortuous pathways could accumulate greater tractography uncertainties during streamline propagation. The observation that regions with greater volume changes exhibited more connectivity alterations suggests that tissue shrinkage is closely associated with connectivity changes, although the precise mechanisms underlying this relationship remain to be elucidated. In addition to geometric changes, altered diffusion properties likely contribute to connectivity changes. Fixation modifies tissue microstructure, changing water diffusion characteristics that form the basis of tractography algorithms [9, 10]. Using diffusion tensor imaging, we previously demonstrated that axial diffusivity, a diffusion tensor metric reflecting water diffusion along the principal fiber direction, is particularly sensitive to regional volume changes following fixation [13]. Although the current study employed CSD-based analysis rather than tensor-based metrics, fixation-induced changes in water diffusion properties would similarly affect fiber orientation distribution estimation. These local diffusion alterations propagate through the tractography pipeline, ultimately affecting connectivity estimates. Both geometric and diffusion-related factors may interact in complex ways, and the relative contribution of each mechanism warrants further investigation.

Beyond these macroscopic geometric and diffusion-related factors, recent work by Santini et al. investigated microstructural changes between in vivo and perfusion-fixed ex vivo marmoset brains using advanced diffusion MRI techniques including oscillating gradient and b-tensor encoding at 9.4T [27]. Their findings demonstrated that perfusion fixation induces substantial microstructural alterations, including decreased extracellular volume fraction, potential axon beading, and increased dot compartment signal fraction. These microstructural changes at the cellular level would further affect fiber orientation distribution estimation and tractography outcomes, complementing the macroscopic tissue deformation discussed above. It should also be noted that the perfusion fixation process itself involves a brief period of blood washout before fixative reaches the tissue, during which transient ischemic conditions may occur. Temporal and frontal regions are known to be particularly vulnerable to ischemic insult due to their vascular anatomy. Although transcardial perfusion in our protocol was performed immediately following deep anesthesia to minimize such effects, we cannot entirely exclude the possibility that perfusion-related tissue changes contributed to the regional patterns of connectivity alteration observed in this study.

### Regional vulnerability

Temporal and limbic regions, including the temporopolar area and hippocampal formation, showed the greatest connectivity changes. These areas contain complex fiber pathways with varying curvature, and such complex fiber architecture may interact with fixation-induced tissue changes to produce greater tractography variability. In contrast, regions with minimal volume changes, such as the piriform cortex and superior colliculus, showed fewer connectivity alterations. This pattern suggests that the extent of local tissue deformation is closely associated with the degree of fixation-induced connectivity changes.

The preferential effect on connections between anatomically distant regions, particularly those involving temporal and frontal areas, further supports the role of geometric factors. Longer pathways traversing multiple brain regions may accumulate greater tractography uncertainties when tissue geometry is distorted.

The observation that connections between neighboring regions showed increased connectivity following fixation may reflect differential effects of tissue shrinkage on local versus distant pathways. Tissue contraction could increase local white matter density, potentially affecting fiber orientation distribution estimation and SIFT2 weighting in ways that differ from longer pathways. However, the precise mechanisms underlying this phenomenon remain unclear and warrant further investigation.

### Implications for ex vivo research

These findings have important implications for the growing field of ex vivo connectomics [5, 6]. First, direct comparison between in vivo and ex vivo tractography should account for systematic connectivity biases, particularly in temporal and limbic networks. Second, cross-species comparisons using ex vivo data may be confounded by differential fixation effects across brain regions. Third, validation of in vivo tractography using ex vivo data requires careful consideration of which connections are most affected by fixation.

The observation that approximately 15% of connections are significantly altered represents a substantial effect that cannot be ignored in ex vivo studies. Researchers using fixed tissue for tractography should consider these biases when interpreting their results.

### Considerations regarding acquisition parameters

It should be noted that the diffusion MRI acquisition parameters differed between in vivo and ex vivo conditions in the present study. These differences were intentional to optimize data quality for each condition, as ex vivo tissue exhibits substantially reduced diffusion coefficients requiring higher b-values for adequate signal detection [19, 20, 28]. Importantly, the ex vivo acquisition employed parameters that are generally more favorable for tractography, including higher angular resolution and finer spatial resolution. Despite these more favorable acquisition conditions, the majority of significantly altered connections showed decreased rather than increased connectivity in the ex vivo condition. This directional pattern suggests that the observed connectivity changes reflect genuine biological effects of fixation rather than artifacts arising from differences in acquisition parameters.

### Limitations

Several limitations should be acknowledged. First, we examined only one fixation protocol using 4% paraformaldehyde, and different fixatives or concentrations might produce different patterns of change. Second, our findings in marmosets may not directly translate to species with different brain sizes or white matter organization. Third, we did not examine the temporal dynamics of connectivity changes during the fixation process. Fourth, while our spatial resolution was high for in vivo standards, subtle connectivity changes might not be detectable at the voxel sizes employed. Fifth, we did not directly quantify geometric changes in white matter pathways such as length and curvature. The extent of fixation-induced geometric deformation likely depends on multiple factors including fixation protocols and species-specific tissue properties. Sixth, in the present study, all tractography analyses were performed in each animal’s native diffusion space, with the atlas parcellation transformed into this space for connectivity matrix construction. An alternative approach would be to register both in vivo and ex vivo data to a common template space to potentially isolate microstructural diffusion changes from macroscopic geometric deformation. However, spatial normalization of diffusion-weighted data introduces interpolation artifacts that contaminate voxel-level diffusion indices through merging and averaging of neighboring voxel information, and this effect becomes more pronounced with larger deformations such as those induced by fixation-related tissue shrinkage. For this reason, performing tractography in native space is the standard approach in diffusion MRI connectivity analysis. Separating the relative contributions of macroscopic geometric changes and microscopic diffusion property alterations remains an important challenge for future studies, potentially through the development of methods that do not require spatial transformation of diffusion data [27, 28].

## Conclusions

Perfusion fixation substantially alters structural brain connectivity in a region-specific manner that strongly correlates with local volume changes. Approximately 15% of inter-regional connections showed significant alterations, with temporal and limbic regions being most affected. Regions with minimal tissue shrinkage showed preserved connectivity patterns. These findings provide crucial reference data for interpreting ex vivo tractography studies and highlight the importance of considering fixation effects in comparative neuroimaging research.

## Supplementary Information

Below is the link to the electronic supplementary material.

Supplementary Material 1

## Data Availability

The datasets generated and/or analyzed during the current study are available from the corresponding author on reasonable request.
