# Supplementary material for: Effects of perfusion fixation on whole-brain structural connectivity in marmoset: a diffusion MRI analysis
Source: Radiol Phys Technol. 2026 Apr 17;19(2):666–73. doi: 10.1007/s12194-026-01036-y (PMC13253769; doi:10.1007/s12194-026-01036-y)
Supplement: Supplementary file 1 — Supplementary Material 1 [file 12194_2026_1036_MOESM1_ESM.docx]

**Effects of perfusion fixation on whole-brain structural connectivity in marmoset: a diffusion MRI analysis**

Supplementary Materials

[Supplementary Table S1. Brain structure atlas 2](#_Toc219299630)

[Supplementary Figure S2. List of nodes associated with each connection that showed a significant increase or decrease 3](#_Toc219299631)

[Supplementary Figure S3. Spatial distribution of connectivity changes shown separately for increased and decreased connections. 4](#_Toc219299632)

# **Supplementary Table S1. Brain structure atlas**

| **Number of regions** | **Region name** |
| --- | --- |
| **1** | **Piriform cortex** |
| **2**  **3** | **Entorhinal cortex** |
| **3** | **Perirhinal cortex** |
| **4** | **Olfactory bulb** |
| **5** | **Olfactory nucleus** |
| **6** | **Frontal pole** |
| **7** | **Orbitofrontal cortex** |
| **8** | **Medial ventral prefrontal cortex** |
| **9** | **Medial prefrontal cortex** |
| **10** | **Dorsolateral prefrontal cortex** |
| **11** | **Ventrolateral prefrontal cortex** |
| **12** | **Premotor** |
| **13** | **primary motor cortex** |
| **14** | **primary somatosensory cortex** |
| **15** | **secondary somatosensory cortex** |
| **16** | **Ventral postal parietal area** |
| **17** | **intraparietal sulcus** |
| **18** | **Postal parietal area** |
| **19** | **Gustatory cortex** |
| **20** | **Temporopolar area** |
| **21** | **Inferior temporal area** |
| **22** | **Mid-temporal area** |
| **23** | **Superior temporal rostral area** |
| **24** | **Superior temporal polysensory cortex** |
| **25** | **Insular cortex** |
| **26** | **Auditory cortex** |
| **27** | **V1** |
| **28** | **V2** |
| **29** | **V3** |
| **30** | **V6** |
| **31** | **Septal nucleus** |
| **32** | **Anterior cingulate** |
| **33** | **Posterior cingulate** |
| **34**  **35**  **36**  **37** | **Retrosplenial cortex** |
| **35** | **Precuneus** |
| **36** | **Parahippocampal gyrus** |
| **37** | **Hippocampal formation** |
| **38** | **Subiculum** |
| **39** | **Amygdala** |
| **40** | **Prostriate area** |
| **41** | **Accumbens nucleus** |
| **42** | **Substantia nigra** |
| **43** | **Bed nucleus of the stria terminalis** |
| **44** | **Caudate nucleus** |
| **45** | **Putamen** |
| **46** | **Claustrum** |
| **47** | **Globus pallidus** |
| **48** | **Medial geniculate nucleus** |
| **49** | **Dorsolateral geniculate nucleus** |
| **50** | **Thalamus** |
| **51** | **Subthalamic nucleus** |
| **52** | **Superior colliculus** |

List of 52 brain regions used for parcellation. Brain regions defined by the marmoset-specific atlas (1).

# **Supplementary Figure S2. List of nodes associated with each connection that showed a significant increase or decrease**

**
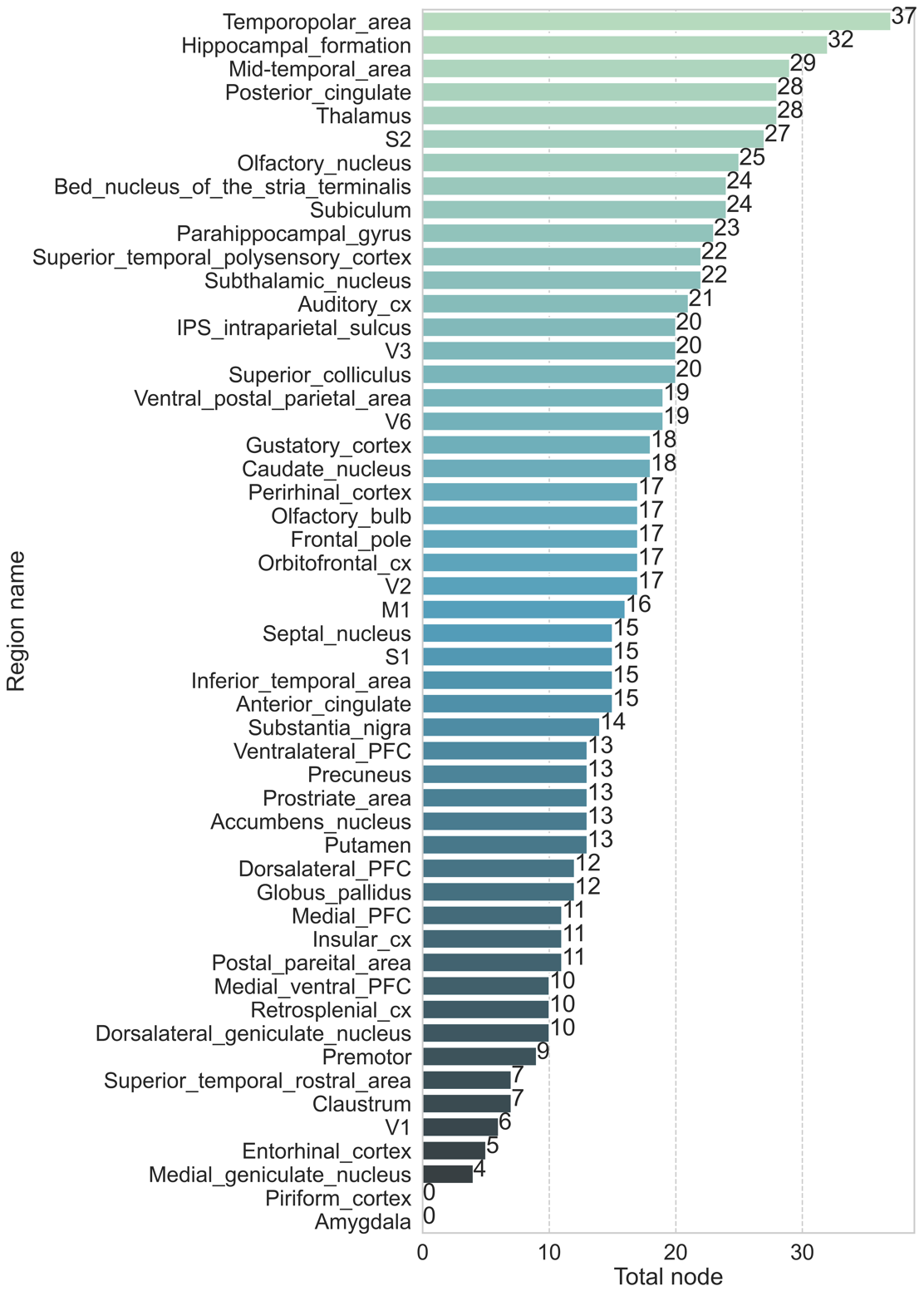
**

The number of significantly changed connections for all 52 brain regions, shown in bar graphs in order of increasing total.

# **Supplementary Figure S3. Spatial distribution of connectivity changes shown separately for increased and decreased connections.**

(A) Connections showing significantly increased connectivity in ex vivo compared to in vivo conditions (red lines). (B) Connections showing significantly decreased connectivity in ex vivo compared to in vivo conditions (blue lines). Both lateral (left) and dorsal (right) views are shown. Green spheres represent brain region centroids.

1. Hashikawa T, Nakatomi R, Iriki A. Current models of the marmoset brain. Neurosci Res. 2015;93:116–127. doi: 10.1016/j.neures.2015.01.009.
